# A pilot study assessing listening comprehension and reading comprehension in children with down syndrome: Construct validity from a multi-method perspective

**DOI:** 10.3389/fpsyg.2022.905273

**Published:** 2022-08-12

**Authors:** Alison Prahl, C. Melanie Schuele

**Affiliations:** ^1^Department of Communication Sciences and Disorders, Baylor University, Waco, TX, United States; ^2^Department of Hearing and Speech Sciences, Vanderbilt University Medical Center, Nashville, TN, United States

**Keywords:** Down syndrome, listening comprehension, reading comprehension, construct validity, psychometrics

## Abstract

Obtaining valid assessments of language and literacy skills in children with Down syndrome (DS) presents a challenge as there is a paucity of information about the psychometrics of measures that are commonly used to measure listening and reading comprehension in this population. Evaluating the construct validity of measures that employ different methods is essential to ascertain the optimal method of assessment in individuals with DS and with typical developmental histories (TD). This pilot study aimed to evaluate the construct validity of four parallel measures of listening and reading comprehension. Participants included 19 individuals with DS (*M* = 17 years, 3 months; *SD* = 3 years, 6 months) and 19 word-level reading-matched children with TD (*M* = 7 years, 2 months; *SD* = 7 months). Participants completed norm-referenced assessments for four parallel measures of listening and reading comprehension. The four measurement methods were: (1) non-verbal response, (2) cloze procedure, (3) passage-level with close-ended questions, and (4) passage-level with open-ended questions. Participants completed additional assessments (e.g., cognition, language, and speech) for descriptive purposes. Construct validity was assessed using the Multitrait-Multimethod Matrix, a correlation matrix arranged to facilitate the assessment and interpretation of construct validity of measures across various formats. For both study groups, we observed strong evidence of construct validity for three out of four measurement methods. Results using the multimethod perspective also indicated that the listening and reading comprehension constructs were not separable. The findings from this pilot study represent a first step toward determining optimal methods of listening and reading comprehension assessment for individuals with DS. Additionally, these results can inform outcome measure selection in future language and literacy research with children with DS.

## Introduction

Down syndrome (DS), the most common known genetic cause of intellectual disability, is characterized by a behavioral phenotype consisting of a pattern of strengths and weaknesses across multiple domains (e.g., cognitive, linguistic, speech-motor, and social-emotion; Chapman and Hesketh, 2000; Fidler, 2005; Fidler and Philofsky, 2009). In relation to reading outcomes, one of the hallmark DS phenotypic characteristics is that individuals often present with language and literacy deficits that are disproportionate to their broader cognitive profiles (Byrne et al., 1995). Although there is substantial literature on language development in children with DS, children and young adults with DS are quite underrepresented in reading research (Afacan et al., 2018). In this study, we aimed to gain insight into evidence-based language and literacy assessment approaches to inform educational and clinical services.

Despite perpetuated beliefs that children with DS cannot learn to read and comprehend text, an emerging body of evidence challenges this assumption (Buckley, 2001; Byrne et al., 2002; Lemons et al., 2017). For example, Buckley (2001) found that 60–70% of individuals with DS in Australia and the United Kingdom have attained functional levels of literacy. Byrne and colleagues found that some children with DS (ages 4–12) demonstrate word-level reading developmental trajectories that are not significantly different compared with development in reading-matched children with typical development (TD; ages 4–10). They also found that children with DS presented with word-level reading standard scores that were higher than their intelligence quotient scores (e.g., Byrne et al., 1995, 2002). Based on current evidence, many individuals with DS present with a relative strength in word-level reading as compared with other reading skills; however, they often experience persistent difficulties with reading comprehension. Research with children and young adults with DS, though limited, demonstrates that reading comprehension growth tends to progress slowly and achievement rarely reaches levels commensurate to word-level reading skills or oral language abilities (e.g., Byrne et al., 2002; Groen et al., 2006; Nash and Heath, 2011).

Reading comprehension–the construction of meaning from written text and the ultimate goal of reading (Catts and Kamhi, 1999)–requires the coordination of multiple underlying cognitive and linguistic processes (Kintsch, 1998; Snow, 2002; Elleman and Compton, 2017; Fuchs et al., 2018). Thus, across multiple theoretical models of reading that place reading comprehension as the outcome of interest, reading comprehension is viewed as a multidimensional construct (Gough and Tunmer, 1986). Within these theoretical models, proficient word recognition (i.e., decoding) and listening comprehension are widely recognized competencies that underlie reading comprehension. Decoding involves context-free word recognition measured by production of real or pseudo words and listening comprehension is the process by which lexical information, sentences, and discourse are interpreted (Gough and Tunmer, 1986). Generally for readers with typical development, once a word is accurately decoded a few times, it is likely to be recognized immediately without any conscious effort, leading to efficient word recognition. As such, across typical development, the influence of word recognition on reading comprehension decreases, whereas the contribution of listening comprehension on reading comprehension increases over time (e.g., Gough and Tunmer, 1986; Catts et al., 2006; García and Cain, 2014; Hogan et al., 2014). Although less is known about the relation between these constructs across development in DS, cross-sectional studies provide useful information. For readers with DS, listening comprehension is reported to predict reading comprehension and is more strongly correlated with reading comprehension in children and young adults with DS than TD peers (e.g., Roch and Levorato, 2009; Prahl and Schuele, 2022). As a result and given that individuals with DS often have a relative strength in word recognition as detected on word recognition tasks rather than decoding tasks (Fidler, 2005; Martin et al., 2009), listening comprehension is hypothesized as the main barrier to reading comprehension. Because individuals with DS often engage in the task of learning to read with difficulties in listening comprehension (Cossu et al., 1993; Roch and Levorato, 2009), evaluating listening comprehension using psychometrically sound measures is an important consideration to understand reading outcomes for individuals with DS. However, it is challenging to obtain valid estimates of these language skills in individuals with DS, as there is limited information specific to DS about the psychometrics of common measures of listening comprehension and reading comprehension. Thus, the purpose of this pilot study was to explore the psychometric properties of commonly used measures of listening comprehension and reading comprehension for individuals with DS.

Three challenges to valid assessment of listening comprehension and reading comprehension include (1) challenges with the constructs, (2) challenges with measures commonly used to assess the constructs of interest, and (3) challenges specific to the DS phenotype. First, given that reading comprehension and listening comprehension are multidimensional constructs, the degree to which measures tap various underlying cognitive and linguistic processes differs based on how listening comprehension or reading comprehension is operationalized with a specific measure. To illustrate the challenges that emerge with measuring reading comprehension, in a study of 97 school-age children with TD, Cutting and Scarborough (2006) found that the relative contributions of word reading (*R*^2^s = 6.1–11.9%) and oral language (*R*^2^s = 9–15%) to reading comprehension varied substantially across three reading comprehension measures: Wechsler Individual Achievement Test (WIAT; Wechsler, 1992), Gates-MacGinitie Reading Test (MacGinitie et al., 2000), and Gray Oral Reading Test (GORT; Wiederholt and Bryant, 1992). Additionally, in their sample of 510 school-age twin sibling pairs with TD, Keenan et al. (2008) found only modest intercorrelations (*rs* = 0.31–0.70) among four commonly used reading comprehension measures: GORT (Wiederholt and Bryant, 1992), Qualitative Reading Inventory (QRI; Lauren and Caldwell, 2001), Woodcock-Johnson Test of Achievement Passage Comprehension subtest (Woodcock et al., 2001), and Peabody Individual Achievement Test Reading Comprehension subtest (Dunn and Markwardt, 1970). Thus, based on these findings, various reading comprehension measures do not seem to be converging on the same construct. Rather, these tests differentially measure the multiple aspects of reading comprehension. Researchers have identified several additional reader characteristics that contribute to comprehending written text, some of which may account for the lack of association across reading comprehension measures (Miller et al., 2013). These characteristics include reading fluency, working memory, verbal reasoning, background knowledge, motivation and engagement, and executive functioning (Perfetti et al., 1996; Snow, 2002; Kintsch and Kintsch, 2005; Cutting and Scarborough, 2006).

It is not surprising that there is similarly a lack of consensus among researchers on how to operationalize listening comprehension and whether listening comprehension and oral language are distinct constructs. Some researchers propose that oral language contributes to listening comprehension, or the opposite, that listening comprehension is part of a broader construct of oral language, and yet others suggest that oral language and listening comprehension are separate constructs (Hogan et al., 2014; Kim and Phillips, 2014; Catts et al., 2015; Gray et al., 2017). In a large-scale longitudinal study designed to increase the field’s understanding of this topic, the Language and Reading Research Consortium, 2017 evaluated the dimensionality of oral language and listening comprehension based on confirmatory factor analysis of data from a population-based sample of preschool through third grade children (*n* = 1,869). Evidence of oral language and listening comprehension operating as a single construct was stronger in the preschool and kindergarten data as compared with the first through third grade data. Although the best fitting model at all grade levels included two separate factors (i.e., expressive and receptive vocabulary and grammar) for oral language and listening comprehension, oral language and listening comprehension were highly correlated (*r* = 0.87–0.91). The LARRC concluded that oral language and listening comprehension were best characterized as a single oral language construct, and thus measures of oral language and listening comprehension were argued to assess the same underlying construct. Based on this conclusion, measures of oral language and measures of listening comprehension can presumably be used interchangeably, as they all would yield an estimate of “listening comprehension.” Given the lack of convergence in measures of reading comprehension and listening comprehension, it is essential that constructs such as these are operationally defined to promote clarity.

For the purpose of the current study, **reading comprehension** was operationalized as constructing meaning from written text and **listening comprehension** was operationalized as constructing meaning from read-aloud written text. These definitions align with how Gough and Tunmer (1986) originally defined reading and listening comprehension within the Simple View of Reading. Gough and Tunmer (1986) further argued that parallel definitions, and thus, parallel measures of listening comprehension and reading comprehension are essential to adequately capturing the relation between these two constructs. Given the parallel nature of the operational definitions, it is not surprising that listening comprehension and reading comprehension have been found to be highly correlated in studies of children with TD and children with DS (Sinatra, 1990; Nation and Snowling, 2004; Roch and Levorato, 2009; Laws et al., 2016). Further, listening comprehension operationalized in this manner is distinct from listening comprehension in conversation or as operationalized in some oral language measures. Unlike listening comprehension as operationalized here, listening comprehension in the context of conversation includes a certain level of redundancy, additional non-verbal cues, and the opportunity to repair any lapses in comprehension that are not available in text. Further, listening comprehension operationalized as such is distinct from other oral language measures (e.g., vocabulary, grammar comprehension) that do not necessarily require text-level processing (Catts and Kamhi, 1986) and instead often involve comprehension of language at the single word or phrase level. Whereas, common measures of oral language (e.g., grammar comprehension and vocabulary) do not have parallel formats with typical measures of reading comprehension, the measures included in this study reflect parallel measures of listening comprehension and reading comprehension that align with the operational definitions above.

Second, listening comprehension and reading comprehension assessment is complicated by substantial variation across measurement methods. To illustrate, Francis et al. (2005) reported a stronger association between decoding and reading comprehension when comprehension was assessed with a cloze-procedure measurement method compared with a multiple-choice question method among children with TD. Commonly used measures vary in the (a) text format that is presented at the single word, phrase, sentence, or paragraph/passage level and (b) response format that requires the test taker, for example, to point to a picture to identify the referent or to verbally answer multiple-choice, close-ended, or open-ended questions. Further, many commonly used standardized measures have psychometric weaknesses for test reliability and validity (Paris and Stahl, 2005). Petersen and Stoddard (2018) argued that because emphasis has been placed on test reliability, many reading comprehension measures with weak validity have emerged. In particular, content validity—how well test items adequately represent the entirety of the measured construct—as well as construct validity—the degree to which a test measures what it claims to be measuring—comes into question. Due to weaknesses in content and construct validity, any conclusions about listening comprehension and reading comprehension must be considered in the context of the specific measure used. For any particular measure of comprehension, it is important to evaluate how the construct is operationalized (e.g., recalling facts and constructing inferences), presentation of the test stimuli (e.g., visual or oral), the response format (e.g., oral or written; multiple-choice; or open-ended), and the test format (e.g., timed or untimed; individual or group administration; Fuchs et al., 2018).

Third, listening comprehension and reading comprehension assessment for individuals with DS warrants careful consideration because most measures were not developed with sufficient attention to the myriad characteristics of individuals with disabilities. Given phenotypic characteristics of DS (e.g., cognitive and linguistic deficits), norm-referenced assessments may not yield valid measurement for this population, despite the demonstration of validity for other populations. In previous studies of reading comprehension in DS, authors do not consistently report reliability scores and validity scores. The DS behavioral phenotype consists of patterns of strengths and challenges across not only cognitive and linguistic domains, but also speech-motor and social-emotional domains. Two challenges characteristic of the DS phenotype, but perhaps not of other groups of individuals with intellectual disabilities, may contribute to underestimation of skills. First, the speech of individuals with DS is characterized by persistent, atypical phonological error patterns that have an adverse impact on intelligibility (Stoel-Gammon, 1997). Reduced speech intelligibility may be a confounding factor for reading comprehension measures requiring a verbal response. Second, when faced with cognitive challenges, individuals with DS are more likely than TD peers to engage in positive and negative behaviors to avoid tasks (Wishart, 1996). This behavior reflects overall poor task persistence and higher levels of off-task social behaviors, especially when cognitive processes are strained, for example, in reading comprehension assessment (Wishart, 1996; Fidler, 2006).

Historically, researchers have not considered behavioral phenotypes in selecting or developing assessment measures to address these challenges (Lemons et al., 2017). Thus, the purpose of this pilot study was to evaluate the validity of listening comprehension and reading comprehension measures for individuals with DS. We evaluated the construct validity, the degree to which a test measures what it claims to be measuring, for four parallel measures of listening comprehension and reading comprehension. The Multitrait-Multimethod matrix (MTMM; Campbell and Fiske, 1959) is an approach using a matrix of correlations to facilitate the assessment and interpretation of the construct validity of measures across various methods. Within the MTMM, convergent validity and discriminant validity is assessed. Convergent validity refers to the degree to which there is empirical evidence that a measure correlates with other measures of the same construct which are assumed to relate based on theory. Discriminant validity refers to the degree to which there is empirical evidence that constructs can be meaningfully differentiated (i.e., not highly correlated) from other theoretically distinct constructs (Campbell and Fiske, 1959). Several traits and several methods are measured and evaluated within the MTMM. In this study, we evaluated two traits—listening comprehension and reading comprehension—and four methods (non-verbal response, cloze-procedure, passage-level with close-ended questions, and passage-level with open-ended questions), resulting in an 8 × 8 matrix.

We addressed two research questions for two groups of participants—individuals with DS and word-level reading-matched children with typical development (TD): (1) For both groups, are measures of the same construct that use different methods (monotrait-heteromethod) more strongly correlated than (a) measures of different constructs that use the same method (heterotrait-monomethod) and (b) measures of different constructs that use different methods (heterotrait-heteromethod)? And (2) Is evidence of construct validity moderated by group?

## Methods

The study procedures were approved by the university Institutional Review Board. The data reported here are part of a study on listening comprehension and reading comprehension in DS (e.g., Hessling, 2020; Prahl and Schuele, 2022). In this article, we present data related to the construct validity and reliability of measures of listening and comprehension in DS and their TD peers.

### Participants and procedure

The study was conducted with two groups in which participants were matched on word-level reading: the first group consisted of 19 individuals with DS (32% boys) ages 10 to 22 years (*M* = 17 years, 3 months; *SD* = 3 years, 6 months) and the second group was comprised of 19 children with TD (42% boys) ages 6–8 years (*M* = 7 years, 2 months; *SD* = 7 months). Because listening comprehension and reading comprehension were the outcomes of interest in this pilot study, participants were matched on the remaining variable most-often included in theoretical models of reading comprehension—word-level reading. To form the TD control group, each participant with DS was matched to one TD participant (i.e., a TD participant could only be paired with a single DS participant) based on word-level reading and sex when possible. A TD child was considered an eligible match if his or her raw score on the Word Identification subtest of the Woodcock Reading Mastery Tests-III (WRMT-III; Woodcock, 2011) was within three points of the raw score for a participant with DS. See Table 1 for participant demographic information. Significant between-group differences were observed on all descriptive measures except word level reading, the matching criteria (see Table 2).

**TABLE 1:** Participant demographic information.

	DS group (*n* = 19)	TD group (*n* = 19)
**Sex**		
Male	8	6
Female	11	13
**Race**		
American Indian/Alaska Native	0	0
Asian	0	0
Black/African American	1	1
Hispanic	0	2
Native Hawaiian/Other Pacific Islander	0	0
White	17	15
Multiple races	1	1
Not reported	0	0
**Ethnicity**		
Hispanic or Latino	1	3
Not Hispanic or Latino	17	15
Not reported	1	1
**Mother’s education level**		
Some high school	0	0
High school diploma/GED	1	0
Some college	2	3
Associate’s degree	3	0
Bachelor’s degree	6	9
Master’s degree	5	4
Professional degree	2	3

This content has been adapted from Prahl and Schuele (2022).

**TABLE 2:** Participant characteristics in raw scores, standard deviations, and ranges.

	DS group (*n* = 19)			TD group (*n* = 19)			
	Mean	SD	Range	Mean	SD	Range	*P*
Age (years; months)	17; 3	3; 6	11; 1–22; 9	7; 2	0; 7	6; 6–8; 1	0.00*
KBIT-2	16.21	5.02	10–28	25.42	5.63	14–34	0.00*
ROWPVT-4	77.58	27.85	22–132	101.47	8.71	82–117	0.00*
EOWPVT-4	82.95	19.40	50–117	96.79	14.32	68–122	0.01*
TACL-4 Grammatical Morphemes	35.53	8.73	19–54	48.16	4.71	41–54	0.00*
WRMT-3 Word Identification	21.32	6.79	12–37	20.84	6.90	11–34	0.83
Arizona-4	88.92	7.27	74–100	97.90	3.34	88–100	0.00*

DS, Down syndrome; TD, Typically developing; SD, Standard deviation; KBIT-2, Kaufman Brief Intelligence Test-Second Edition (Kaufman, 2004); ROWPVT-4, Receptive One Word Picture Vocabulary Test-Fourth Edition (Martin and Brownell, 2011b); EOWPVT-4, Expressive One Word Picture Vocabulary Test-Fourth Edition (Martin and Brownell, 2011a); TACL-4, Test of Auditory Comprehension of Language-Fourth Edition (Carrow-Woolfolk, 2014); *TACL-4 Scaled scores not reported for DS Group because the age range of the DS group extended beyond the TACL-4 normative age; WRMT-III, Woodcock Reading Mastery Tests-Third Edition (Woodcock, 2011); Arizona-4, Arizona Articulation Phonology Scale-Fourth Edition (Fudala and Stegall, 2017). This content has been adapted from Prahl and Schuele (2022).

Participants with DS were recruited by distributing study flyers at private schools, on electronic mailing lists, and with DS community organizations in the Nashville, TN and Dallas/Fort Worth, TX regions as well as with families whose children had participated in previous research studies in the lab. Participants with TD were recruited solely from the Nashville, TN metropolitan area by distributing flyers on electronic mailing lists, to families whose children had participated in previous research studies in the lab, to community organizations, and families of local elementary school first- and second-grade students who were reading on grade level. Participants were compensated $15 for completing the eligibility session and $40 for completing the assessment session as well as an additional $20 if participants traveled to the university lab to complete the study activities.

Individuals with DS were eligible to participate if they (a) had been diagnosed with DS by a physician per parent report, (b) were monolingual English speakers and used spoken language as a primary form of communication, (c) successfully completed the screening battery (i.e., listened to directions, completed assessments), and (d) had normal or corrected-to-normal vision per parent report. Hearing status inclusionary criteria was not used for the DS group to ensure inclusion of a representative sample of participants with DS, who frequently present with mild-to-moderate hearing loss (Roizen et al., 1993). Children with TD were eligible to participate if they (a) demonstrated oral language skills within normal limits (i.e., standard score = 85) and neurotypical development per parent report; (b) were monolingual English speakers; (c) successfully completed the screening battery (i.e., listened to directions, completed assessments); (d) passed hearing screening in at least one ear, unaided using American Speech-Language-Hearing Association (ASHA) (2022); and (e) had normal or corrected-to-normal vision per parent report. Exclusionary criteria for both groups included correctly reading fewer than 80% of words on the Phonological Awareness Literacy Screening—Kindergarten (PALS-K; Invernizzi et al., 1997) and children with TD were excluded if they scored more than 1.5 standard deviations below the mean on the measure of non-verbal cognition. Seven consented individuals with DS were not eligible to participate; one individual did not successful complete the screening battery, and six individuals did not meet the word reading criteria. Five consented children with TD were not eligible to participate; four did not meet the word reading criteria, and two were not monolingual English speakers.

Participants completed two individual sessions (eligibility and assessment) at the university lab, school, community location (e.g., public library), or in their home. Parents or guardians provided written consent (or participants/power of attorneys for individuals over the age of 18), and participants provided written assent. Each participant’s guardian provided demographic background information by completing an intake questionnaire. Eligibility measures included a hearing screening (for the TD group only), word-level reading screening, and measure of non-verbal cognition. To match participants in the TD and DS groups, a word-level reading measure was also administered during the eligibility session. Additional descriptive measures administered at the eligibility session included measures of oral language (receptive and expressive vocabulary and grammar comprehension) and speech accuracy. All eligibility session measures were administered in the same fixed order. The eligibility session lasted 45–60 min. The first author, a certified speech-language pathologist, collected all study data. See Tables 2, 3 for participant raw scores and standard scores, respectively, on the descriptive measures.

**TABLE 3:** Participant characteristics in standard score or scaled score means, standard deviations, and ranges.

	DS group (*n* = 19)			TD group (*n* = 19)		
	Mean	SD	Range	Mean	SD	Range
KBIT-2	52.37	12.25	40–80	109.47	13.26	82–127
ROWPVT-4	59.63	7.87	55–81	112.89	7.80	96–127
EOWPVT-4	62.67	10.81	55–86	111.32	14.57	85–131
TACL-4 grammatical morphemes*				11.58	2.22	8–15
WRMT-3 word identification	61.68	11.07	55–86	110.21	15.91	75–138
Arizona-4	57.5	15.82	50–96	99.58	1.16	96–100

DS, Down syndrome; TD, Typically developing; SD, Standard deviation; KBIT-2, Kaufman Brief Intelligence Test-Second Edition (Kaufman, 2004); ROWPVT-4, Receptive One Word Picture Vocabulary Test-Fourth Edition (Martin and Brownell, 2011b); EOWPVT-4, Expressive One Word Picture Vocabulary Test-Fourth Edition (Martin and Brownell, 2011a); TACL-4, Test of Auditory Comprehension of Language-Fourth Edition (Carrow-Woolfolk, 2014); *TACL-4 Scaled scores not reported for DS Group because the age range of the DS group extended beyond the TACL-4 normative age; WRMT-III, Woodcock Reading Mastery Tests-Third Edition (Woodcock, 2011); Arizona-4, Arizona Articulation Phonology Scale-Fourth Edition (Fudala and Stegall, 2017). This content has been adapted from Prahl and Schuele (2022).

Assessment measures included four methods of measuring listening comprehension and four methods of measuring reading comprehension. The selected methods represent a range of text and response formats that may frequently be encountered in academic and vocational settings (see Table 4). The specific measures were selected because the initial test items at lower levels of difficulty and complexity and the amount of scaffolding provided (i.e., illustrated items on the WRMT-III Passage Comprehension subtest, non-verbal response required on the KABC Reading/Understanding subtest) were expected to reduce task demands to minimize floor effects. Assessment order was counterbalanced across participants in each group to control for order effects. Participants were given breaks between tasks as needed to maintain attention and on-task behavior. The assessment session for each participant lasted 75–100 min. All eligibility and assessment measures were administered in accordance with the manualized directions unless otherwise noted.

**TABLE 4:** Methods of measuring listening comprehension and reading comprehension.

Method	Text format	Response format	Listening comprehension measure	Reading comprehension measure
Non-verbal response	Phrase and sentence	Non-verbal (pointing, acting out)	WJ IV Test of Oral Language Understanding Directions subtest	KABC Reading/Understanding subtest
Cloze-procedure	Sentence and paragraph	Verbal, one word	WJ IV Test of Oral Language Oral Comprehension subtest	WRMT-III Passage Comprehension subtest
Passage-level with close-ended questions	Paragraph	One word, verbal or pointed	TILLS Listening Comprehension subtest	TILLS Reading Comprehension subtest
Passage-level with open-ended questions	Paragraph	Verbal	WIAT-III Listening Comprehension subtest	WIAT-III Reading Comprehension subtest

WJ IV, Woodcock-Johnson IV (Schrank et al., 2014); KABC, Kaufman Assessment Battery for Children (Kaufman and Kaufman, 1983); WRMT-III, Woodcock Reading Mastery Tests–Third Edition (Woodcock, 2011); TILLS, Test of Integrated Language and Literacy (Nelson et al., 2015); WIAT-III, Wechsler Individual Achievement Test–Third Edition (Wechsler, 2009). This content has been adapted from Prahl and Schuele (2022).

### Measures

#### Descriptive measures

##### Hearing screening

Pure tone audiometry with a standard hand-raising response was used to screen hearing acuity in both ears at frequencies of 500, 1,000, 2,000, and 4,000 Hz at 30 dB. For the DS group, when a participant failed to respond to a particular frequency at 30 dB, the intensity of the tone was increased until a reliable response was obtained for descriptive purposes. The highest intensity necessary to elicit a passing response (two out of three presentations) was recorded. The participants with DS’ responses to the tones ranged from 30 to 70 dB (*M* = 35, *SD* = 10) at 500 Hz, 30–60 dB (*M* = 34, *SD* = 9) at 1,000 Hz, 30–55 dB (*M* = 32, *SD* = 6) at 2,000 Hz, and 30–70 dB (*M* = 36, *SD* = 11) at 4,000 Hz.

##### Non-verbal intelligence

The Kaufman Brief Intelligence-Second Edition Matrices subtest (KBIT-2; Kaufman, 2004) was administered as a measure of non-verbal intelligence. Test takers infer a relation or rule in a set of pictures or patterns and point to the picture or pattern that best fits the relation or rule. The KBIT-2 includes simple oral instructions and only requires test takers to answer with a meaningful gesture such as pointing. The K-BIT is normed for individuals ages 4–90 and is ideal for those with limited language ability. The mean internal-consistency reliability by age was 0.88 and the mean test-retest reliability by age was 0.83, as reported in the K-BIT manual.

##### Oral language

The Receptive and Expressive One Word Picture Vocabulary Tests-Fourth Editions (ROWPVT-4 and EOWPVT-4; Martin and Brownell, 2011a,b) were administered as measures of receptive and expressive semantic knowledge. For the ROWPVT-4, test takers point to the picture (out of a field of four) that corresponds with the word the examiner says aloud. The ROWPVT-4 manual reported median internal consistency reliability coefficient by age of 0.97 and the test-retest reliability coefficient of 0.97. For the EOWPVT-4, test takers name pictures. The EOWPVT-4 manual reported median internal consistency reliability coefficient by age of 0.95 and the test-retest reliability coefficient of 0.98. These measures are normed for individuals ages 2–70. The Test of Auditory Comprehension of Language-Fourth Edition Grammatical Morphemes subtest (TACL-4; Carrow-Woolfolk, 2014) was administered as a measure of grammar comprehension. Test takers point to the picture (out of a field of three) that corresponds to stimuli of increasing grammatical complexity presented orally by the examiner. The TACL-4 is normed for individuals ages 3–12. Due to limited grammar comprehension characteristic of the DS phenotype, participants with DS did not reach ceiling levels on this measure despite that the DS participant age range extended beyond the normative age range. The TACL-4 manual reported Grammatical Morphemes mean internal consistency reliability of 0.95 and test-retest reliability of 0.71. The TACL-4 is a valid measure of oral language based on strong evidence of content-description, criterion-prediction, and construct-identification validity.

##### Word-level reading

On the PALS-K primer list (eligibility measure), test takers read a list of 20 isolated, real words. Each word read accurately via decoding or automatic recognition is scored as correct; percent correct was calculated. On the WRMT-III Word Identification subtest test takers read isolated, real words. A word is scored correct if read accurately within approximately 5 s, whether it is decoded or automatically recognized. In addition to participant matching, the WRMT-III Word Identification raw scores and standard scores are reported for descriptive purposes. Each DS participant began reading at one of the first three entry points depending on the ease with which they read the PALS-K words and each TD participant began reading at their respective grade level entry point. The manualized instructions were then followed to establish the basal and ceiling. The manual reported mean internal-consistency reliability by school-level socioeconomic status of 0.93 and the mean test-retest reliability by age of 0.92. In addition to participant matching, the WRMT-III Word Identification raw scores and standard scores are reported for descriptive purposes. The WRMT-III is normed for individuals ages 4; 6–79. The manual reported mean split-half reliability coefficient by age of 0.93 and the test-retest reliability coefficient of 0.95 for pre-kindergarten through Grade 2, 0.90 for Grades 3–8, and 0.88 for Grades 9–12.

##### Speech

The Arizona Articulation and Phonology Scale-Fourth Revision (Arizona-4; Fudala and Stegall, 2017) was administered as a measure of speech accuracy. Test takers label pictures. If the child does not provide the intended label, the label is modeled by the examiner and repeated by the test taker. The examiner notes speech sound production errors. The Word Articulation Total Score was calculated based on the weighted values (a reflection of how frequently the sound occurs in American speech) of the sounds that were produced accurately. The Arizona-4 is normed for individuals ages 18 months to 21 years. Internal consistency coefficients reported in the manual ranged from 0.90 to 0.97 depending on age and test-retest reliability was 0.96. The Arizona-4 has strong evidence of content, response process, construct, and convergent validity.

#### Dependent variable measures

##### Listening comprehension

Raw scores were calculated for all four listening comprehension measures to capture incremental differences between participants that would be obscured by using standard scores for individuals with DS (Mervis and Klein-Tasman, 2004). Two subtests from the Woodcock-Johnson IV Test of Oral Language (WJ IV; Schrank et al., 2014), normed for individuals ages 2–90 years, were administered. The Understanding Directions subtest requires a non-verbal response. Test takers follow single-sentence directions, presented orally via an audio recording to point to familiar objects with varying characteristics (e.g., size and location) in a picture scene. This subtest has a median reliability of 0.86 in the 5–19 age range and 0.87 in the adult age range as reported in the manual. The Oral Comprehension subtest uses a cloze procedure. Test takers listen to a short audio-recorded passage and supply the missing word from the final sentence in a one- or two-sentence passage. This subtest has a median reliability of 0.82 in the 5–19 age range and 0.80 in the adult age range. The Test of Integrated Language and Literacy Skills (TILLS; Nelson et al., 2015). Listening Comprehension subtest was administered as a measure that used passage-level text paired with close-ended questions. It is normed for individuals ages 6;0 to 18;11. On this subtest, test takers selected “yes,” “no,” or “maybe” to answer questions about passage-level text read aloud by the examiner. As an accommodation, a card with the three choices (yes, no, and maybe) was placed on the table in front of the examiner as additional visual support and to provide a non-verbal response option. The mean intraclass reliability coefficient reported in the manual was 0.95 and test-retest reliability was 0.77. The TILLS was found to have strong construct and concurrent validity. The Wechsler Individual Achievement Test-III (WIAT-III; Wechsler, 2009) Listening Comprehension Oral Discourse Comprehension subtest was administered. Test takers listen to audio-recorded passage-level text and then verbally answer open-ended questions read aloud by the examiner. Test takers’ answers were scored according to the possible correct answers listed on the Record Form; one point was awarded for each correct answer and zero points for incorrect answers. The mean internal reliability coefficient reported in the manual was 0.83 and test-retest reliability was 0.75. The WIAT-III was found to have strong evidence of validity based on content, response process, and internal structure.

##### Reading comprehension

The Kaufman Ability Battery for Children (KABC; Kaufman and Kaufman, 1983) Reading/Understanding subtest requires a non-verbal response. Test takers act out written directions. The Reading/Understanding subtest is normed for individuals ages 7–12. The manual reported mean internal consistency coefficient based on the split-half reliability method based on age of 0.90 for preschool children and 0.93 for children ages 5–12 years and the test-retest reliability coefficient of 0.83, 0.88, and 0.92 for ages 2; 6–4, 5–8, and 9–12; 6, respectively. The WRMT-III Passage Comprehension subtest uses cloze procedure. Initial passages are single sentences and passages increase in length across the subtest. Initial passages are accompanied by a picture, but pictures are phased out as passages increase in length. Test takers supply the missing word located anywhere in the passage to complete the meaning of a sentence or paragraph that they read. The manual reported mean internal consistency coefficient based on the split-half reliability method based on age of 0.90 and the test-retest reliability coefficient of 0.86 for Pre-Kindergarten-Grade 2, 0.88 for grades 3–8, and 0.81 for grades 9–12. Raw scores were calculated on the KABC Reading/Understanding and WRMT-III Passage Comprehension subtests. The TILLS Reading Comprehension subtest was administered as a measure that used passage-level text paired with close-ended questions. It is normed for individuals ages 6; 6–18; 11. On this subtest, test takers read passage-level text and questions and then selected “yes,” “no,” or “maybe” to answer the questions. In accordance with the manualized directions, the TILLS Reading Comprehension subtest was discontinued if test takers made seven or more miscues when reading the first passage. Rather than assigning a raw score of 0, for the purpose of this study, if the discontinue rule was met, the participant was considered to score at the floor level and a score was not included on this measure for the construct validity analyses. The mean intraclass reliability coefficient reported in the manual was 0.99 and test-retest reliability was 0.86. The WIAT-III Reading Comprehension subtest uses passage-level text paired with open-ended questions. Test takers read passage-level text and then verbally answer open-ended questions read aloud by the examiner. Test takers’ answers were scored according to the criteria provided on the Record Form; answers could be scored as 2-points, 1-point, or 0-points for some questions and scored as 2-point or 0-points on other questions. Four to eight questions were asked per passage. For participants with DS, the entry point was based on their word-level reading grade equivalent based on the WRMT-III Word Identification subtest and for TD participants, the entry point was based on their current grade level. Because WIAT-III Reading Comprehension scores are based on the particular item set administered and the total raw scores from different item sets are not directly comparable, vertically scaled scores (i.e., weighted scores) were used as outlined in the assessment manual. The WIAT-III is normed for individuals ages 4–50. The mean internal reliability coefficient reported in the manual was 0.86 and test-retest reliability was 0.90.

### Design and variables

**FIGURE 1:**
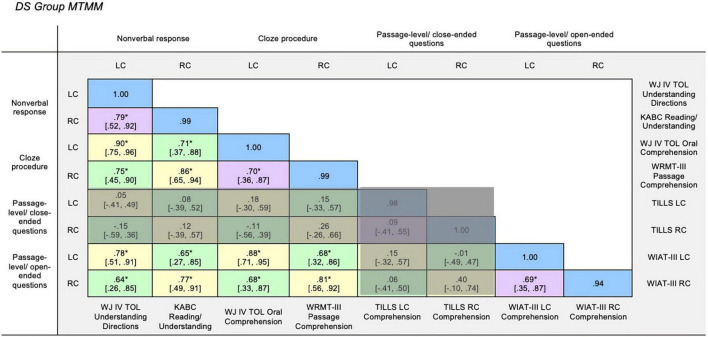
DS group MTMM. Multitrait-multimethod matrix for Down syndrome group. Monotrait-monomethod cells (reliability diagonal) marked in blue, monotrait-heteromethod cells (validity diagonal) marked in yellow (heterotrait-monomethod cells marked in purple, and heterotrait-heteromethod cells marked in green. LC, Listening Comprehension; RC, Reading Comprehension; WJ IV TOL, Woodcock-Johnson Test of Oral Language IV (Schrank et al., 2014); KABC, Kaufman Assessment Battery for Children (Kaufman and Kaufman, 1983); WRMT-III, Woodcock Reading Mastery Tests-Third Edition (Woodcock, 2011); WIAT-III, Wechsler Individual Achievement Test-Third Edition (Wechsler, 2009). *p > 0.05.

**FIGURE 2:**
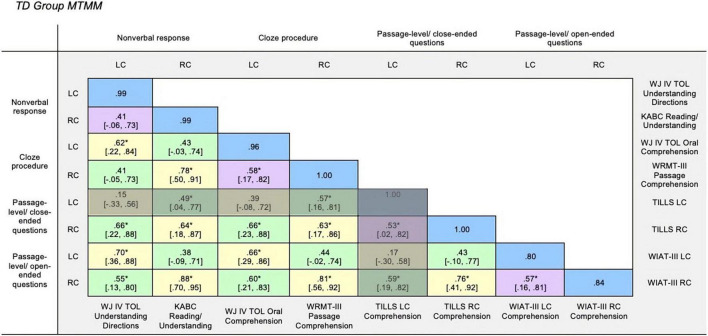
TD group MTMM. Multitrait-multimethod matrix for Down syndrome group. Monotrait-monomethod cells (reliability diagonal) marked in blue, monotrait-heteromethod cells (validity diagonal) marked in yellow, heterotrait-monomethod cells marked in purple, and heterotrait-heteromethod cells marked in green. LC, Listening Comprehension; RC, Reading Comprehension; WJ IV TOL, Woodcock-Johnson Test of Oral Language IV (Schrank et al., 2014); KABC, Kaufman Assessment Battery for Children (Kaufman and Kaufman, 1983); WRMT-III, Woodcock Reading Mastery Tests-Third Edition (Woodcock, 2011); WIAT-III, Wechsler Individual Achievement Test-Third Edition (Wechsler, 2009). *p > 0.05.

To establish inter-rater reliability, initially the first author scored all measures. A graduate student reliability coder with formal training in psychoeducational assessment was trained on the scoring procedures for the dependent measures. She then independently scored the participants’ assessment sessions from video and audio recordings for a random selection (=25%) of participants; only video recordings with camera angles that allowed for valid assessment scoring were eligible for random selection. Inter-rater reliability was estimated using intraclass correlation coefficients (ICCs). ICCs account for differences in scores between coders as well as the variance among participants on the measures of interest. For the dependent measures, the mean ICC value was 0.99 for the DS group and 0.93 for the TD group (Hessling, 2020) and thus, the primary coder’s scoring was used in the analyses. The ICC values were all excellent for the DS group (0.94–1.00) and the values ranged from good to excellent for the TD group (0.80–1.00). For both groups, the lowest ICC values were observed for the WIAT-III measures which is not surprising given that the response format is an open-ended verbal response, and thus the rubric requires decisions by the coder which can lead to lack of agreement across scorers. See the blue cells in Figures 1, 2 for the ICC values for each measure by group. The primary scorer and reliability scorer double scored all measures from the assessment protocols (93% inter-rater agreement) and discrepancies were resolved by consensus before data was double entered for analysis.

To answer research question one, separate MTMM were created for the DS and TD groups and analyzed based on Campbell and Fiske’s (1959) guidelines. Within the matrices, four classes of cells are distinguished. *Monotrait-monomethod cells* (reliability diagonal, blue cells) constitute the main diagonal of the matrix and contain the reliability coefficient of each trait in each method, as measured by interclass correlations as an estimate of inter-rater reliability. Because a high consistency of scores is an essential requirement for test validity, the monotrait-monomethod cells are expected to be the highest values in the MTMM. *Monotrait-heteromethod cells* (validity diagonal, yellow cells) reflect the correlation between measures of the same trait measured using different methods (convergent validity). Because the two measures are of the same trait, strong correlations are expected. *Heterotrait-monomethod cells* (purple cells) reflect the correlation among measures that share the same measurement method, but measure different traits. These values are considered an index of discriminant validity and thus should be weaker than the correlations in the yellow cells. If, however, these correlations are high, it is because measuring different constructs with the same methods results in correlated measures. *Heterotrait-heteromethod cells* (green cells) reflect the correlation among measures that differ in trait and method (discriminant validity). Because these correlations share neither trait nor method, the heterotrait-heteromethod cells are expected to be the lowest values in the MTMM. The degree to which these cells are smaller than the heterotrait-monomethod cells is considered an index of the influence of the methods factor. Summary level statistics are reported for each matrix to ascertain the extent to which the cells overlap or differ from one another according to Campbell and Fiske’s (1959) guidelines. To determine whether the correlations were significantly different, we evaluated whether there was overlap in the confidence intervals around the correlation coefficients. In addition, we demonstrated sufficient power (=0.80) to interpret at least moderate correlation coefficients (0.50–0.70) within the MTMM using G*Power 3.1 Software (Faul et al., 2009). Cook’s distance was used to monitor for undue influence across analyses relevant to each cell within the MTMM. There was no evidence that any individual data points were leveraging regression lines. Because scores were not reported for participants who met the discontinue rule on the TILLS Reading Comprehension measure, follow-up analyses demonstrated that the study results were robust to listwise deletion. To answer the second research question, a combined MTMM with data from both groups was created. We conducted 56 separate regression analyses for each cell to evaluate whether the evidence of construct validity was moderated by group for each cell in the MTMM. In addition to monitoring the data for outliers, scores for both groups were determined to be normally distributed based on visual analysis of histograms. For each regression, we were interested in evaluating the interaction effect—whether the effect of one measure on another measure changed depending on group membership (DS vs. TD).

## Results

Mean listening comprehension and reading comprehension raw scores as well as the number of participants who completed the measures and yielded scores above the floor level (i.e., raw score > 0) are displayed in Table 5.

**TABLE 5:** Participant reading comprehension and listening comprehension raw scores and participants scoring above floor level.

	TD group (*n* = 19)				DS group (*n* = 19)			
Measure	Mean	SD	Range	# (%) of participants above floor level	Mean	SD	Range	# (%) of participants above floor level
**Listening comprehension**								
WJ-IV TOL Understanding Directions	35.47	6.53	22–50	19 (100)	17.74	9.89	2–37	19 (100)
WJ-IV TOL Oral Comprehension	15.26	2.62	10–20	19 (100)	7.84	4.62	0–17	17 (89)
TILLS Listening Comprehension	13.26	3.87	7–20	19 (100)	9.05	3.55	0–15	18 (95)
WIAT-III Listening Comprehension	11.42	1.90	8–16	19 (100)	4.95	4.13	0–15	18 (95)
**Reading comprehension**								
KABC Reading/Understanding	10.58	5.64	2–19	19 (100)	8.58	5.32	0–18	17 (89)
WRMT-III Passage Comprehension	13.32	3.73	9–22	19 (100)	8.68	4.41	2–17	19 (100)
TILLS Reading Comprehension	9.53	3.80	4–15	15 (79)	5.12	3.82	0–11	16 (84)
WIAT-III Reading Comprehension*	46.37	9.71	30–64	19 (100)	27.42	14.67	2–55	19 (100)

*Vertically scaled scores (not raw scores) reported for this measure, due to administration rules.

TD, typically developing; DS, Down syndrome; SD, standard deviation; WJ IV, Woodcock-Johnson IV (Schrank et al., 2014); KABC, Kaufman Assessment Battery for Children (Kaufman and Kaufman, 1983); WRMT-III, Woodcock Reading Mastery Tests–Third Edition (Woodcock, 2011); TILLS, Test of Integrated Language and Literacy (Nelson et al., 2015); WIAT-III, Wechsler Individual Achievement Test–Third Edition (Wechsler, 2009).

### Evaluating construct validity in Down syndrome group

The DS group MTMM is displayed in Figure 1. The reliability diagonal marked in blue reflects the interclass correlation values as a measure of inter-rater reliability for each measure. The interclass correlation values for all measures were excellent (ICCs = 0.94–1.00).

#### Monotrait-heteromethod

The monotrait-heteromethod cells marked in yellow reflect the correlation between measures of the same trait using different measurement methods. Statistically significant and strong correlations (*rs* = 0.77–0.90, *p* < 0.05) were observed in half of the monotrait-heteromethod yellow cells, thus reflecting good convergent validity across the measures except for the passage-level with close-ended questions (TILLS) measures. Two notable exceptions of non-significant and weak correlations were observed, first, for the relation between the passage-level with close-ended questions (TILLS) listening comprehension measure (*rs* = 0.05–0.18, *p* > 0.05) and each of the other measures. Second, non-significant and weak correlations were observed for the relation between the passage-level with close-ended questions (TILLS) reading comprehension measure (*rs* = 0.12–0.40, *p* > 0.05) and each of the other measures. Given that the TILLS measures are not converging with other measures of the same construct, it appears that the TILLS measures are not tapping the construct that it’s purporting to measure. It also may be the case that the TILLS measures are tapping a different dimension of the construct when compared with the other measurement methods. Because of the questionable construct validity of the TILLS (shaded cells in Figure 1), we will hone in on the cells reflecting only the associations of the remaining measures from this point forward.

#### Heterotrait-monomethod

The heterotrait-monomethod cells marked in purple reflect the correlations between listening comprehension and reading comprehension measures using the same method. Statistically significant and strong correlations (*rs* = 0.69–0.79, *p* < 0.05) were observed between the two traits—listening comprehension and reading comprehension—for three out of the four measurement methods (i.e., non-verbal response, cloze procedure, and passage-level with open-ended questions). This pattern of strong correlations (Cohen, 1998) demonstrates shared method variance, that measuring different constructs with the same methods results in correlated measures. The values in monotrait-heteromethod (yellow) cells were not significantly stronger than the values in the heterotrait-monomethod (purple) cells, as evidenced by the overlapping confidence intervals.

#### Heterotrait-heteromethod

Lastly, the heterotrait-heteromethod cells marked in green reflect the correlation between different traits measured using different methods. When excluding the associations related to the TILLS measures, statistically significant and strong correlations (*rs* = 0.64–0.75, *p* < 0.05) were observed in the remaining heterotrait-heteromethod green cells. Based on the overlapping confidence intervals, the values in the heterotrait-heteromethod (green) cells were not all significantly weaker than the values in the heterotrait-monomethod or the monotrait-heteromethod cells.

#### Summary of construct validity in Down syndrome group

In summary, the results for the DS group provide some support that these various measurement methods for listening comprehension and reading comprehension, with the exception of the TILLS measures, are measuring the same constructs. Within the DS group, however, the results do not provide evidence of discriminant validity. In other words, the listening comprehension and reading comprehension constructs are not separable.

### Evaluating construct validity in typical development group

The TD group MTMM is displayed in Figure 2. Similar to the DS group, the interclass correlation values for all measures ranged from good to excellent (ICCs = 0.80–1.00) as shown in the reliability diagonal marked in blue.

#### Monotrait-heteromethod

The monotrait-heteromethod cells marked in yellow reflect the correlation between measures of the same trait using different measurement methods. Statistically significant and strong (*r* = 0.5; Cohen, 1998) correlations (*rs* = 0.62–0.88, *p* < 0.05) were observed in 75% of the monotrait-heteromethod yellow cells, thus reflecting good convergent validity across all measures. Similar to the DS group, though only for the passage-level with close-ended questions (TILLS) listening comprehension measure, non-significant and weak correlations (*rs* = 0.15–0.39, *p* > 0.05) were observed between this measure and each of the other measures. Given that this listening comprehension measure was not converging with other measures of the same construct, it appears that the TILLS listening comprehension measure is not tapping the construct that it purports to measure for the TD group. Because of the questionable construct validity of the TILLS listening comprehension measure (shaded cells in Figure 2), we again hone in on the cells reflecting only the associations of the remaining measures from this point forward.

#### Heterotrait-monomethod

The heterotrait-monomethod cells marked in purple reflect the correlations between listening comprehension and reading comprehension measures using the same method. Statistically significant and strong correlations (*rs* = 0.53–0.58, *p* < 0.05) were observed between the two traits of interest—listening comprehension and reading comprehension—for three out of the four measurement methods (i.e., cloze procedure, passage-level with close-ended questions, and passage-level with open-ended questions). However, the two traits of interest were not significantly correlated (*r* = 0.41, *p* > 0.05) for the non-verbal response (KABC Reading/Understanding and WJ IV TOL Oral Comprehension) measurement method. This pattern of strong correlations (Cohen, 1998) demonstrates shared method variance, that measuring different constructs with the same methods results in correlated measures. It is also important to note that the values in monotrait-heteromethod (yellow) cells were not significantly stronger than the values in the heterotrait-monomethod (purple) cells, as evidenced by the overlapping confidence intervals.

#### Heterotrait-hetermethod

Lastly, the heterotrait-heteromethod cells marked in green reflect the correlation between different traits measured using different methods. Statistically significant and strong correlations (*rs* = 0.49–0.66, *p* < 0.05) were observed in 58% of the heterotrait-heteromethod green cells, with the remaining cells reflecting moderate correlations (*rs* = 0.38–0.44, *p* > 0.05). Given this range of values and the overlapping confidence intervals, the values in the heterotrait-heteromethod (green) cells were not all significantly weaker than the values in the heterotrait-monomethod or the monotrait-heteromethod cells.

#### Summary of construct validity in typical development group

In summary, the results in the TD group provide some support that these measures, with the exception of the TILLS Listening Comprehension measure, are measuring the same constructs. Within the TD group, the results do not provide evidence of discriminant validity. In other words, listening comprehension and reading comprehension constructs are not separable.

### Construct validity group comparisons

**FIGURE 3:**
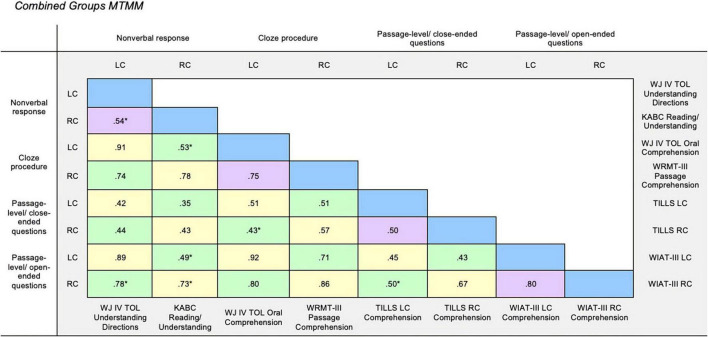
Combined groups MTMM. Multitrait-multimethod matrix for Down syndrome group. Monotrait-monomethod cells (reliability diagonal) marked in blue, monotrait-heteromethod cells (validity diagonal) marked in yellow, heterotrait-monomethod cells marked in purple, and heterotrait-heteromethod cells marked in green. LC, Listening Comprehension; RC, Reading Comprehension; WJ IV TOL, Woodcock-Johnson Test of Oral Language IV (Schrank et al., 2014); KABC, Kaufman Assessment Battery for Children (Kaufman and Kaufman, 1983); WRMT-III, Woodcock Reading Mastery Tests-Third Edition (Woodcock, 2011); WIAT-III, Wechsler Individual Achievement Test-Third Edition (Wechsler, 2009). *p > 0.05.

Regression analyses were performed to test whether the associations of interest within the MTMM (excluding the reliability diagonal) varied according to group. Only five associations were significantly different, all but one of which were within heterotrait cells. See Figure 3 and Table 6 for the regression results. Four associations reflected that correlations were slightly stronger in the DS group, although all correlations for both groups ranged from moderate to strong (*rs* = 0.38–0.79). Further, these correlations did not yield a meaningful interpretation given that they all index associations between indices purported to tap different constructs (i.e., associations moderated by group were all in heterotrait cells). The remaining three correlations moderated by group suggest that associations were attenuated in the DS group compared with the TD group. However, two of these correlations were expected to be small given that they were values contained in heterotrait-heteromethod cells. That is, they reflect correlations between variables that were purported to tap different constructs using different methods. In summary, only a few associations were moderated by group and thus the moderated associations on the whole do not suggest variable construct validity for these measures in the TD group compared with the DS group. The pattern of results was not moderated in any way that is meaningful for interpretation within the MTMM.

**TABLE 6:** Regression coefficients predicting each listening comprehension and reading comprehension measure.

													Passage-level/						Passage-level/					
Variable	Non-verbal response						Cloze procedure						Multiple-choice questions						Open-ended questions					
	LC: WJ TOL understanding directions			RC: KABC reading/understanding			LC: WJ TOL oral comprehension			RC: WRMT passage comprehension			LC: TILLS listening comprehension			RC: TILLS reading comprehension			LC: WIAT listening comprehension			RC: WIAT reading comprehension		
								
	Est.	*SE*	*P*	Est.	*SE*	*P*	Est.	*SE*	*P*	Est.	*SE*	*P*	Est.	*SE*	*P*	Est.	*SE*	*P*	Est.	*SE*	*P*	Est.	*SE*	*P*
Predicting non-verbal response LC: WJ TOL understanding directions																								
Measure × Group				1.22	0.39	0.00*	0.63	0.51	0.22	1.05	0.52	0.05*	−0.33	0.84	0.69	−1.29	0.79	0.11	0.30	0.86	0.73	0.17	0.23	0.46
Predicting non-verbal response RC: KABC reading/understanding																								
Measure × Group	−0.12	0.22	0.60				−0.04	0.48	0.93	−0.03	0.29	0.91	−0.16	0.50	0.75	−0.70	0.45	0.13	−0.13	0.70	0.86	−0.22	0.12	0.07
Predicting Cloze Procedure LC: WJ TOL Oral Comprehension																								
Measure × Group	0.09	0.12	0.46	0.52	0.22	0.02*				0.37	0.27	0.18	−0.06	0.39	0.87	−0.60	0.37	0.12	0.20	0.35	0.57	0.08	0.10	0.45
Predicting cloze procedure RC: WRMT passage comprehension																								
Measure × Group	−0.15	0.17	0.39	0.14	0.19	0.45	−0.36	0.35	0.32				−0.19	0.39	0.62	−0.36	0.36	0.33	−0.32	0.53	0.56	−0.12	0.09	0.21
Predicting passage-Level/MC LC: TILLS listening comprehension																								
Measure × Group	−0.028	0.21	0.20	−0.08	0.27	0.78	−0.55	0.41	0.19	−0.23	0.32	0.48				−0.43	0.34	0.22	−0.28	0.58	0.63	−0.18	0.12	0.16
Predicting passage-level/MC RC: TILLS reading comprehension																								
Measure × Group	−0.54	0.20	0.01*	−0.38	0.26	0.15	−1.06	0.40	0.01*	−0.39	0.32	0.23	−0.44	0.36	0.23				−0.89	0.58	0.13	−0.21	0.11	0.07
Predicting passage-level/OE LC: WIAT listening comprehension																								
Measure × Group	0.10	0.13	0.44	0.40	0.20	0.06	0.33	0.21	0.12	0.40	0.25	0.11	0.13	0.33	0.70	−0.23	0.32	0.49				0.10	0.09	0.30
Predicting passage-level/OE LC: WIAT reading comprehension																								
Measure × Group	−0.18	0.52	0.72	0.42	0.58	0.47	−0.16	0.98	0.87	0.48	0.67	0.48	−0.60	1.10	0.59	−0.39	0.96	0.69	−0.37	1.42	0.80			

*p > 0.05.

WJ TOL, Woodcock-Johnson IV Test of Oral Language (Schrank et al., 2014); WIAT, Wechsler Individual Achievement Test–Third Edition (Wechsler, 2009); TILLS, Test of Integrated Language and Literacy (Nelson et al., 2015); KABC, Kaufman Assessment Battery for Children (Kaufman and Kaufman, 1983); WRMT, Woodcock Reading Mastery Tests–Third Edition (Woodcock, 2011); Est., Estimate; MC, Multiple-Choice; OE, Open-Ended.

## Discussion

In this pilot study, we assessed the construct validity of four parallel measures of listening comprehension and reading comprehension for individuals with DS and their peers with TD. Evaluation of psychometric properties is important to validate the use of commonly used norm-referenced measures for various clinical populations. Though establishing measures as demonstrating strong reliability and validity is essential in development, it is also essential to determine whether those characteristics hold true for each research sample of interest. Further, given that there is a variety of methods for assessing listening comprehension and reading comprehension, it is important to determine whether measures of the same traits using different methods demonstrate convergent validity (Cutting and Scarborough, 2006; Keenan et al., 2008). Researchers can make informed decisions regarding assessment and outcome measure selection based on the empirical evidence regarding feasibility and psychometric properties of commonly used measures.

### Demonstrating construct validity

The MTMM approach proposed by Campbell and Fiske (1959) was chosen for the assessment and interpretation of construct validity. We were interested in evaluating whether measures of the same construct that use different methods were more strongly correlated than (a) measures of different constructs that use the same method and (b) measures of different constructs that use different methods. In other words, we were interested in evaluating whether the monotrait-heteromethod (yellow) associations were more strongly correlated compared with the heterotrait-monomethod (purple) and heterotrait-heteromethod (green) associations. Inspection of the MTMMs for both groups revealed that monotrait-heteromethod associations were not significantly different when compared with the heterotrait-monomethod and heterotrait-heteromethod associations, as evidenced by overlapping confidence intervals. Thus, the results indicate that the listening comprehension and reading comprehension constructs may not be separable or cannot be meaningfully differentiated for the study groups in this developmental period using these particular measures.

The current preliminary findings are consistent with the broader literature in which researchers have suggested that listening comprehension and reading comprehension are highly intercorrelated in readers (e.g., Sticht et al., 1974; Sinatra, 1990; Nation and Snowling, 2004). In a study of concurrent and longitudinal predictors of reading comprehension, Nation and Snowling (2004) examined reading development in 72 children at 8.5 and 13 years of age. Based on concurrent analyses at Time 1, they found that even after controlling for non-verbal cognition, phonological awareness, semantics, and expressive vocabulary, listening comprehension was the strongest contributor to reading comprehension, accounting for 31% of the unique variance. Based on longitudinal analyses, they found that even after controlling for Time 1 non-verbal cognition, reading comprehension, non-word reading, phonological awareness, semantics, and expressive vocabulary, listening comprehension accounted for an additional 14% of the unique variance in reading comprehension at Time 2. Further, Ebert and Scott (2016) found statistically significant and strong correlations between listening comprehension and reading comprehension among younger (*r* = 0.47, *p* < 0.05; aged 6.0–8.11) and older (*r* = 0.47, *p* < 0.05; aged 9.1–16.7) school-aged children with TD. Listening comprehension and reading comprehension have been found to be highly intercorrelated (*rs* = 0.41–56, *p* < 0.05) in studies with children with DS as well, with some stronger correlations between listening comprehension and reading comprehension observed in children with DS compared with children with TD (Roch and Levorato, 2009; Laws et al., 2016). Our results indicate a similar pattern with stronger correlations between listening comprehension and reading comprehension measured using the same method observed in the DS group (*rs* = 0.69–0.79) compared with the TD group (*rs* = 0.41–0.58).

It is important to consider the possible influences of development when interpreting these findings. Based on the simple view of reading model, it is not surprising that we observed strong intercorrelations between listening comprehension and reading comprehension given that the participants in this study had achieved some level of proficiency with word recognition. However, the strength of the relation between listening comprehension and reading comprehension is likely to vary across development. Thus, as other researchers have suggested, measuring listening comprehension earlier in development may be useful in predicting future reading comprehension (Nation and Snowling, 2004; Ebert and Scott, 2016). Capturing the predictive power of listening comprehension may be particularly important when children are developing reading skills or may be considered emergent readers, which may be a prolonged process for individuals with DS. Additionally, despite establishing strong correlations between listening comprehension and reading comprehension, the constructs are not perfectly correlated, thus some unexplained variance remains. Although beyond the scope of this project, future research must evaluate how other variables, such as those illustrated in Scarborough’s (2001) reading rope model, contribute to reading comprehension for individuals with DS.

Inspection of the MTMMs provides additional information regarding the construct validity of the measurement methods (e.g., non-verbal response, cloze response, passage-level text with closed-ended response, and passage-level text with open-ended response) as well as the specific measures evaluated in this study. For the TD and DS groups, the results reflect high inter-rater reliability (ICC > 0.5) which is a precursor to further evaluate validity. For the DS group, the pattern of results reflecting high monotrait-heteromethod correlations (*r* = 0.5) were strongly in favor of convergent validity for three listening comprehension and reading comprehension measures. Three of the measurement methods converged on single listening comprehension and reading comprehension constructs: non-verbal response, cloze procedure, passage-level with open-ended questions. These results could also be interpreted as demonstrating that the three specific measures administered for each of these methods converged on single listening comprehension and reading comprehension constructs. For the TD group, the pattern of results reflecting high monotrait-heteromethod correlations (*r* = 0.5) were strongly in favor of convergent validity for all but one measure. Three of the measurement methods converged on a single listening comprehension construct: non-verbal response, cloze procedure, passage-level with open-ended questions and all of the measurement methods converged on a single reading comprehension construct.

Although the passage-level with close-ended questions (TILLS) listening comprehension and reading comprehension measures reflected a high degree of inter-rater reliability, inspection of the MTMM provides no evidence of construct validity, with one exception in the TD group. The correlations related to indices that were derived for the TILLS measures did not reflect strong construct validity for the DS group (TILLS Listening Comprehension and Reading Comprehension subtests) and was replicated in the TD group (TILLS Listening Comprehension subtest). Next, we speculate about some possible explanations for why the construct validity of the TILLS measure may be compromised in our study sample.

One possible explanation relates to the response format of the TILLS—passage-level text with close-ended questions. Anecdotally, some participants appeared to randomly select a response given the close-ended or forced choice comprehension questions which in turn is not necessarily a true reflection of their listening comprehension or reading comprehension. Another possible explanation related to the response format is the presence of “maybe” as a potential answer choice for the close-ended questions. Being able to consider “maybe” to a comprehension question reflects a certain degree of abstraction, which participants may not have fully understood, despite completing trial items with instructional feedback provided for the “maybe” response, if needed. We acknowledge that we did not account for a number of other variables that may influence performance on these measures. For instance, perhaps the TILLS measures, when used with children in these age and developmental ranges, are influenced more by verbal working memory or place greater demands on the decoding skills of participants when compared with the other measurement methods. For example, in the TD group, one possible explanation for why differential evidence of construct validity was observed may be that the TILLS Listening Comprehension measure was more heavily influenced by verbal working memory compared with the TILLS Reading Comprehension measure. The higher memory load required for the TILLS passage-level text measures may also explain why this measure did not converge with the other listening comprehension and reading comprehension measures in the DS group. Additional research evaluating the construct validity of the TILLS for individuals with DS is also warranted.

### Group comparisons

In comparing the DS and TD groups, we aimed to evaluate the extent to which the evidence of construct validity was moderated by group. Within the MTMMs, a similar pattern of construct validity was demonstrated between groups, and thus only 7 or 25% of associations were moderated by group, though not in any particularly meaningful way. We only evaluated whether the associations were moderated by group because we hypothesized that group membership would capture any other potential moderators given that group differences on any other variables (e.g., non-verbal cognition and grammar comprehension) would be accounted for by group membership.

### Limitations

The results of this study should be interpreted with the following limitations in mind. First, the MTMM approach that we used involves a primarily logical rather than analytical approach to guide interpretation of construct validity. Despite this limitation, analyzing and reporting the confidence intervals within the MTMMs enabled us to evaluate the extent to which the various classes of cells differed from one another according to Campbell and Fiske’s (1959) guidelines. Further analysis using confirmatory factor analysis was not possible in this pilot study, though this approach could be used to evaluate construct validity with a larger participant sample. Second, we acknowledge that not all individuals with DS demonstrate sufficient word-level reading and reading comprehension skills necessary to complete the reading and language-related literacy tasks included in this study. In the current study, six individuals with DS (10 to 18 years of age) did not meet the eligibility criteria to participate in the study due to limited word-level reading abilities. These individuals may have presented with some pre-reading skills, though they did not demonstrate sufficient reading given the established eligibility criteria and thus we cannot draw any conclusions regarding the reading abilities for those non-readers or emergent readers who were not included in the study. Further, the study results should be viewed as a minimal estimate of the construct validity of the measures given that convenience sampling was used and due to the small sample size. In summary, the study results are specific to a particular subset of individuals with DS and the degree to which these results can be generalized for a broader and more representative sample in individuals with DS is unknown.

Finally, we did not control for a number of variables that are known to contribute to listening comprehension and reading comprehension in the analyses evaluating whether the evidence of construct validity was moderated by group. As mentioned previously, we hypothesized that any between-group differences that may have moderated the evidence of construct validity would be accounted for in analyzing the effect of group membership. To evaluate this hypothesis, future research should explore the extent to which participant characteristics such as working memory, background knowledge, verbal reasoning, and executive functioning influence the construct validity of various measurement methods across clinical populations (e.g., Perfetti et al., 1996; Snow, 2002; Kintsch and Kintsch, 2005; Miller et al., 2013).

### Implications

The methods employed in this study address many of the challenges to valid assessment of listening comprehension and reading comprehension for individuals with DS. Although challenges with how listening comprehension and reading comprehension are defined will likely persist, we have clearly operationalized these two constructs as well as selected measures that align with those definitions. Reading comprehension was operationalized as constructing meaning from written text, and listening comprehension was operationalized as constructing meaning from read-aloud written text. In addition, the various measurement methods included in this study reflect a comprehensive range of methods for assessing listening comprehension and reading comprehension. Lastly, we also considered the DS phenotype in selecting which measures to evaluate. Given that floor effects are often observed when using norm-referenced assessments with individuals with DS, we included measures with a range of text formats so that participants, specifically those with limited word-level reading, would be able to complete at least some initial test items. Further, because individuals with DS often have limited speech intelligibility, we included measures with a range of response formats to limit the impact of poor speech intelligibility on examiner understanding and scoring of responses. We demonstrated a high degree of inter-rater reliability for the measurement methods that required a non-verbal or minimal verbal response. Not surprisingly we observed the lowest degree of inter-rater reliability in both groups, though still an acceptable value, for the most verbally robust (passage-level text with open-ended question) measurement method.

Consistent with the rationale for conducting this study, the results provide guidance on potentially valid measures for assessing listening comprehension and reading comprehension for individuals with DS and their peers with TD. Overall, the results demonstrate the inter-rater reliability of all the measures evaluated. However, strong evidence of convergent validity was only observed for three out of the four measurement methods, with no evidence of discriminant validity for the listening comprehension and reading comprehension constructs. The construct validity results are of critical importance in regards to using psychometrically sound assessment measures. Further, for examiners who may not have experience assessing and making accuracy judgements for individuals with limited speech intelligibility, it may be important to consider the response format alongside the evidence presented herein. Similarly, for test takers with limited reading proficiency, it also may be important to consider the text format. Taken together, the study results can guide listening comprehension and reading comprehension assessment selection for individuals with DS and their peers with TD. By establishing the inter-rater reliability and construct validity of multiple listening comprehension and reading comprehension measurement methods, researchers and clinicians can have greater confidence in using these measures to quantify skills and characterize patterns of strengths and weaknesses.

### Future directions

This initial measurement investigation lays the foundation for developing and evaluating individualized reading interventions for individuals with DS. The current study results provide preliminary evidence of construct validity of multiple measurement methods, as well as identified the optimal methods of assessment, inform the outcome measure selection for studies of reading intervention for individuals with DS. Future analyses of the data from this investigation will apply generalizability (G) theory to conduct a decision (D) study to determine the number of measures needed to obtain stable estimates of listening comprehension and reading comprehension based on the current data of individuals with DS. The results of a G and D study will extend the current findings and enable researchers to ascertain how many of the evaluated (valid) measures should be administered to adequately capture an individual’s listening comprehension and reading comprehension skills. As mentioned previously, additional research is needed for a larger sample replication and with individuals across different stages of reading development. Future research should also further evaluate the construct validity of the passage-level with close-ended questions (TILLS) listening comprehension and reading comprehension measures.

### Conclusion

The current study contributes to the evidence base regarding the reliability and validity of commonly used measures of listening comprehension and reading comprehension in terms of their utility for individuals with DS. Key findings include strong evidence of reliability and construct validity for three of four measurement methods (non-verbal response, cloze procedure, and passage-level with open-ended questions). These results support the use of these measurement methods in clinical practice and future studies of reading comprehension in individuals with DS.

## Data availability statement

The raw data supporting the conclusions of this article will be made available by the authors, without undue reservation.

## Ethics statement

The studies involving human participants were reviewed and approved by Vanderbilt University Institutional Review Board. Written informed consent to participate in this study was provided by the participants’ legal guardian/next of kin.

## Author contributions

AP: conceptualization, formal analysis, investigation, project administration, and writing and preparing manuscript for sending. CS: conceptualization, supervision, reviewing, and editing manuscript. Both authors contributed to manuscript revision, read, and approved the submitted version.
